# Alleged Extirpation of the Liver

**Published:** 1847-12

**Authors:** 


### ALLEGED EXTIRPATION OF THE LIVER.

We have been shown, by a friend, a letter from Dr. Robert Thompson, of Ohio, which states, that on the 10th ult. (Nov.) he extirpated the enlarged LIVER of a woman, of Licking county, Ohio; and, that a day and a half after the operation, the patient was doing well! We of course supposed the letter to be a forgery; but a few days afterwards, the fact was announced in the newspapers of that quarter, and, subsequently, the gentleman, to whom we have referred, received, as he informs us, a second letter, dated *eight* days after the operation, when the patient was still alive. In one of the newspaper accounts it is stated, that the tumor, "independent of the fluid it contained, weighed 29½ pounds. It *proved* to be the whole liver, which had undergone degeneration and had become filled with hydatids." From the word which we have underscored, we infer, that before and during the operation, the tumor was not regarded as consisting of, or comprising, the liver; and, therefore, that the operator and the respectable gentleman who assisted him did not plan the extirpation of that organ. But *was* it extirpated? The decision that it was, seems to have been made from an inspection of the mass after it was taken out, and if the gall bladder were in it, the conclusion must be received; but no other evidence would establish the fact, seeing that the structure was morbid and filled with hydatids. We may presume, then, that the gentlemen who made the decision had the proof to which we have referred. Now, after discovering that the liver had been, inadvertently, removed, on what ground could they expect the recovery of the patient? What anatomist has yet detected the veins by which the blood of the cæliac and superior mesenteric arteries could find its way back to the vena cava? And if it could not return, how could death be averted? But if a venous anastomosis existed, which might supply the place of the vena portæ and hepatic veins, what surgeon would expect to see them unite, and become obliterated, under the ligature? Finally, who would look for a union of the sides of the common gall duct, so as to prevent regurgitation from the duodenum into the peritoneal sac? With the thoughts suggested by these questions, we may well be surprised that the patient should be alive eight days after the operation. This, if the organ really were extirpated, may, perhaps, be explained by a reference to the entire emaciation and consequent anemic condition in which the patient was, as we have been informed, at the time of the operation.

For ourselves, however, until shown convincing evidence that the excised mass was, or included, the liver, we shall regard it of a different kind, and believe that the organ, in a state of atrophy, from the pressure of the tumor, was pushed far up in the concave of the diaphragm, to which it might morbidly adhere, and thus escaped the knife of the intrepid operator. If not hepatic, the tumor might have been encephaloid or ovarian, most likely the latter; for, as we learn from the friend to whom we have already referred, the uterus, as he has been informed, was enlarged, hardened, pressed low into the pelvis, and partially retroverted. We shall keep this case in view, and give our readers a further account of it in our next number.

